# Activation of calcitonin gene-related peptide signaling through the prostaglandin E2-EP1/EP2/EP4 receptor pathway in synovium of knee osteoarthritis patients

**DOI:** 10.1186/s13018-016-0460-4

**Published:** 2016-10-17

**Authors:** Atsushi Minatani, Kentaro Uchida, Gen Inoue, Shotaro Takano, Jun Aikawa, Masayuki Miyagi, Hisako Fujimaki, Dai Iwase, Kenji Onuma, Toshihide Matsumoto, Masashi Takaso

**Affiliations:** 1 Department of Orthopedic Surgery, Kitasato University School of Medicine, 1-15-1 Minami-ku Kitasato, Sagamihara City, Kanagawa 252-0374 Japan; 2 Department of Pathology, Kitasato University School of Medicine, 1-15-1 Minami-ku Kitasato, Sagamihara City, Kanagawa 252-0374 Japan

**Keywords:** Knee osteoarthritis, Calcitonin gene-related peptide, Cyclooxygenase-2

## Abstract

**Background:**

Calcitonin gene-related peptide (CGRP) is a 37-amino-acid vasodilatory neuropeptide that binds to receptor activity-modifying protein 1 (RAMP1) and the calcitonin receptor-like receptor (CLR). Clinical and preclinical evidence suggests that CGRP is associated with hip and knee joint pain; however, the regulation mechanisms of CGRP/CGRP receptor signaling in synovial tissue are not fully understood.

**Methods:**

Synovial tissues were harvested from 43 participants with radiographic knee osteoarthritis (OA; unilateral Kellgren/Lawrence (K/L) grades 3–4) during total knee arthroplasty. Correlationships between the mRNA expression levels of CGRP and those of tumor necrosis factor-α (TNF-α), interleukin (IL)-1β, IL-6, and cycloxygenase-2 (COX-2) were evaluated using real-time PCR analysis of total RNA extracted from the collected synovial tissues. To investigate the factors controlling the regulation of CGRP and CGRP receptor expression, cultured synovial cells were stimulated with TNF-α, IL-1β, IL-6, and prostaglandin E2 (PGE2) and were also treated with PGE2 receptor (EP) agonist.

**Results:**

CGRP and COX-2 localized in the synovial lining layer. Expression of COX-2 positively correlated with CGRP mRNA expression in the synovial tissue of OA patients. The gene expression of CGRP and RAMP1 increased significantly in synovial cells exogenously treated with PGE2 compared to untreated control cells. In cultured synovial cells, CGRP gene expression increased significantly following EP4 agonist treatment, whereas RAMP1 gene expression increased significantly in the presence of exogenously added EP1 and EP2 agonists.

**Conclusions:**

PGE2 appears to regulate CGRP/CGRP receptor signaling through the EP receptor in the synovium of knee OA patients.

## Background

The main symptom of knee osteoarthritis (OA) is joint pain. Nonsteroidal anti-inflammatory drugs (NSAIDs) are the most widely used pharmaceuticals for treating OA [1]. Although NSAIDs are effective for reducing pain [2], drugs in this class are nephrotoxic and significantly increase the risk of gastrointestinal ulceration and bleeding and cardiovascular events [3]. For these reasons, the long-term use of NSAIDs is contraindicated in many OA patients, and a need therefore exists for the development of more effective and specific drugs for OA pain management.

Calcitonin gene-related peptide (CGRP) is a 37-amino-acid vasodilatory neuropeptide that binds to receptor activity-modifying protein 1 (RAMP1) and the calcitonin receptor-like receptor (CLR) [4]. Clinical and preclinical evidence suggests that CGRP is associated with hip and knee joint pain [5–14]. For example, CGRP-positive cells were immunohistochemically detected in the synovial tissue of OA patients [8, 11]. CGRP mRNA was also observed in the synovial tissue of developmental dysplasia of the hip patients [10], and the mRNA and protein expression of CLR and RAMP1 were detected in cultured synovial cells harvested from OA patients [5]. In addition, CGRP antagonist administration to rat OA models led to the relief of pain [6, 7]. Taken together, these observations suggest that CGRP/CGRP receptor signaling in synovial tissue plays an important role in OA pathology. However, the regulatory mechanisms of CGRP and its receptor in synovial tissue are not fully understood.

Here, we investigated the regulation of CGRP and the CGRP receptor in the synovium of 43 knee OA patients.

## Methods

### Reagents

Human recombinant interleukin-6 (IL-6), interleukin-1β (IL-1β), and tumor necrosis factor-α (TNFα) were purchased from Biolegend (San Diego, CA, USA). Prostaglandin E2 (PGE2) and butaprost (EP2 agonist) were purchased from Sigma (St. Louis, MO, USA). Iloprost (EP1 agonist), sulprostone (EP3 agonist), and CAY10598 (EP4 agonist) were purchased from Caymann (Ann Arbor, MI, USA).

### Patients

A total of 43 participants with radiographic knee OA (unilateral Kellgren/Lawrence (K/L) grades 3–4) underwent total knee arthroplasty at our institution. The study included 9 men and 34 women aged 50–87 years (mean ± SD, 73.6 ± 8.7 years) with a mean ± SD body mass index (BMI) of 26.1 ± 3.9 kg/m^2^ (range 20.3–36.7). The K/L grades of the 43 operated knees were comprised of 42 % grade 3 and 58 % grade 4. A sample of synovial tissue was harvested from each operated knee during the total knee arthroplasty surgery. A portion of each synovial tissue sample was fixed with 4 % paraformaldehyde formalin for 48 h for histological analysis, and the remaining sample was stored at −80 °C until used for RNA extraction for real-time PCR analysis. Synovial tissues from 12 patients were also used for cell culture. Informed consent for participation in this study was obtained from each patient on the day before surgery.

### Immunohistochemistry

To determine the localization of CGRP and cyclooxgenase 2 (COX-2), the paraformaldehyde-fixed synovial tissues samples were embedded in paraffin and sliced into 3-μm-thick sections. Sections were immunohistochemically stained with rabbit polyclonal primary antibody against COX-2 (Abcam, Cambridge, MA) or mouse monoclonal primary antibody against CGRP (Abcam) using the streptavidin-biotin-peroxidase method (Histofine SAB-PO Kit; Nichirei, Tokyo, Japan).

### Real-time PCR analysis

Total RNA was isolated from the harvested synovial tissue using TRIzol reagent (Invitrogen, Carlsbad, CA) following the manufacturer’s protocol. The extracted RNA was used as template for first-strand cDNA synthesis of CGRP, RAMP1, CLR, COX-2, TNF-α, IL-1β, and IL-6 using SuperScript III RT (Invitrogen) in reaction mixtures composed of 2 μL cDNA, 0.2 μM specific primer pair, 12.5 μL SYBR *Premix Ex Taq* (Takara, Kyoto, Japan), and nuclease-free water in a final volume of 25 μL. The primers were designed using Primer Blast software and were synthesized by Hokkaido System Science Co., Ltd. (Sapporo, Japan). The sequences of the PCR primer pairs are listed in Table 1. The specificity of the amplified products was examined by melt curve analysis. Quantitative PCR was performed using a Real-Time PCR Detection System (CFX-96; Bio-Rad, CA, USA) to determine relative mRNA expression levels. The PCR cycle parameters were as follows: 95 °C for 1 min, followed by 40 cycles of 95 °C for 5 s and 60 °C for 30 s. mRNA expression was normalized to the levels of GAPDH mRNA.

**Table 1:** Sequences of the primers used in this study

Primer	Sequence (5′–3′)	Product size (bp)
CGRP-F	TTGCCCAGAAGAGAGCCTGTG	91
CGRP-R	TTGTTCTTCACCACACCCCCTG	
Cox-2-F	TGGCTGAGGGAACACAACAG	74
Cox-2-R	AACAACTGCTCATCACCCCA	
IL-6-F	GAGGAGACTTGCCTGGTGAAA	199
IL-6-R	TGGCATTTGTGGTTGGGTCA	
IL-1β-F	GTACCTGTCCTGCGTGTTGA	153
IL-1β-R	GGGAACTGGGCAGACTCAAA	
TNF-α-F	CTTCTGCCTGCTGCACTTTG	118
TNF-α-R	GTCACTCGGGGTTCGAGAAG	
RAMP1-F	GGCCTCTGGCTGCTCCTG	172
RAMP1-R	GCTCCCTGTAGCTCCTGATG	
CLR-F	TGCAAGACCCCATTCAACAAG	70
CLR-R	TTCCAGCAGAGCCATCCATC	
GAPDH-F	TGTTGCCATCAATGACCCCTT	202
GAPDH-R	CTCCACGACGTACTCAGCG	

### Synovial cell culture

To investigate the factors regulating CGRP and CGRP receptor expression, synovial cells were harvested from synovium collected from the knees of 12 OA patients. Mononuclear cells were isolated from synovium by digestion of the tissue with 40 ml of 0.1 % type I collagenase. The obtained cells were cultured in α-MEM in 6-well plates. After 7 days, the cells harvested from six patients were stimulated with human recombinant IL-6 (100 ng/ml), IL-1β (50 ng/ml), TNF-α (10 ng/ml), or PGE2 (10 μM) for 24 h. The cells harvested from another six patients were stimulated with 10 μM iloprost, butaprost, sulprostone, or CAY10598 for 24 h. After the treatments, cells were harvested for RNA isolation, as described above, and CGRP, RAMP1, and CLR expression was analyzed by RT-PCR. Cells were also harvested for protein extraction, as described below, and RAMP1 protein expression was analyzed by Western blotting.

### Western blotting

To investigate RAMP1 protein expression, cells harvested from five patients were stimulated with 10 μM PGE2, iloprost, butaprost, sulprostone, or CAY10598 for 24 h. Synovial cells were then lysed in RIPA buffer (Wako) supplemented with a protease inhibitor cocktail (Roche), and the protein concentration for each tissue extract was determined using the bicinchoninic acid (BCA) assay (Pierce, Rockford, IL, USA). Protein extracts (10 μg/lane) were separated by sodium dodecyl sulfate-polyacrylamide gel electrophoresis and were electrophoretically transferred onto polyvinyl difluoride membranes, which were then blocked with PVDF blocking reagent (Toyobo, Osaka, Japan) for 1 h. The blocked membranes were incubated overnight at 4 °C with rabbit monoclonal primary antibodies against RAMP1 (Abcam). The primary antibodies were diluted 1:1000 with Can Get Signal Solution 1 (Toyobo). The membranes were washed with PBS-T and incubated with the secondary antibodies (GE Healthcare, NJ, USA), which were diluted 1:1000 with Can Get Signal Solution 2 (Toyobo). Immunoreactive proteins were visualized by chemiluminescence using ImmunoStar LD reagent (Wako, Tokyo, Japan), and images were captured using a LAS-5000 system (Fuji Film, Tokyo, Japan).

### Statistical analysis

Pearson’s correlation coefficient was used to evaluate the relationship between CGRP and the examined stimulatory factors. A *p* value of <0.01 was considered statistically significant for the correlation coefficient analysis. Differences between the untreated and treated synovial cells were compared using one-way ANOVA with Fisher’s least significant difference test. A *p* value of <0.05 was considered statistically significant. Cook’s distance statistical test was used to identify potential statistical outliers that influence the linear regression coefficient analysis. All statistical analyses were performed using SPSS software (v. 19.0; SPSS, Chicago, IL, USA).

## Results

### Relationship between CGRP, inflammatory cytokines, and COX-2 expression levels in synovial tissue of OA patients

**Fig. 1:**
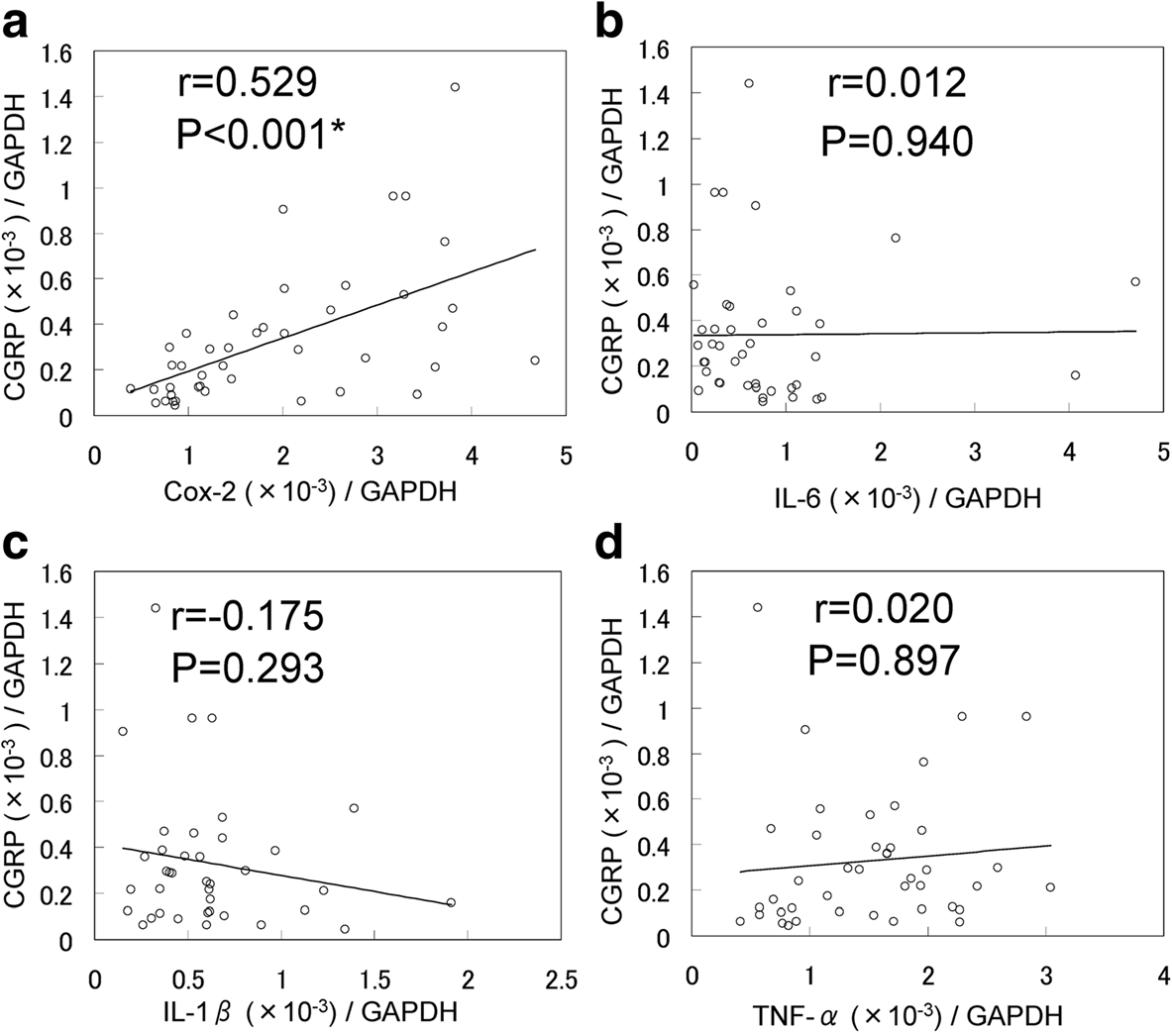
Correlation between mRNA expression levels of CGRP and those of inflammatory cytokines and COX-2 in synovial tissue. The correlation between CGRP and Cox-2 (**a**), IL-6 (**b**), IL-1β (**c**), and TNF-α (**d**) mRNA expression levels in synovial tissue harvested from 43 knee OA patients. *Pearson’s coefficient *p* < 0.001

Seven synovial tissue samples had seven outliers (two for IL-6 and five for IL-1B), which were excluded from the analysis. The relationship between CGRP mRNA expression and the mRNA expression levels of several inflammatory factors in synovial tissue of OA patients was first examined. The expression levels of cyclooxgenase 2 (COX-2) were positively correlated with those CGRP, whereas no correlation between the mRNA expression levels of IL-6, IL-1β, or TNF-α and those of CGRP were detected in the synovial tissue (Fig. 1).

### Localization of COX-2 and CGRP in synovial tissue of OA patients

**Fig. 2:**
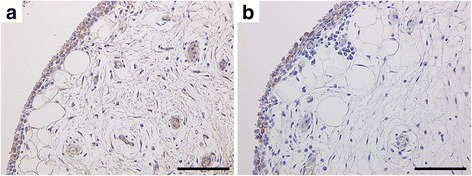
Immunolocalization of CGRP and COX-2 in synovial tissue. Immunolocalization of CGRP (**a**) and Cox-2 (**b**). *Scale bar* = 100 μm

As real-time PCR analysis detected a correlation between CGRP and COX-2 mRNA expression, immunohistochemical analysis was performed to investigate the localization of CGRP and COX-2. The analysis revealed that both COX-2 and CGRP protein localized in the synovial lining layer (Fig. 2).

### Effect of inflammatory cytokines and PGE2 on CGRP expression in cultured synovial cells

**Fig. 3:**
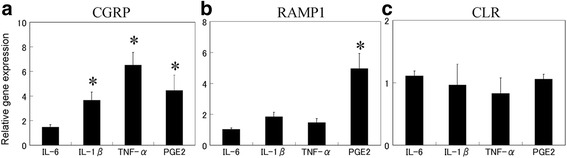
Effects of cytokines and PGE2 on CGRP and CGRP receptor expression in synovial cell culture. Real-time polymerase chain reaction analysis for **a** calcitonin gene-related peptide (*CGRP*), **b** receptor activity-modifying protein 1 (*RAMP1*), and **c** calcitonin receptor-like receptor (*CLR*) gene expression in synovial cell culture. Synovial cells were stimulated with human recombinant IL-6 (100 ng/ml), IL-1β (50 ng/ml), TNF-α (10 ng/ml), or PGE2 (10 μM) for 24 h prior to the extraction and analysis of total RNA. All data are presented as the mean ± standard error (*n* = 6). *p < 0.05 compared with the untreated control

The effects of inflammatory cytokines and PGE2 on the mRNA expression of CGRP and its associated receptors in synovial tissue of the knee OA patients were next examined. As TNF-α and IL-1β stimulate CGRP expression in several cell lines in vitro [15, 16], these inflammatory cytokines were used as positive controls. Real-time PCR analysis revealed that the gene expression of CGRP increased significantly in synovial cells in the presence of exogenously added TNF-α, IL-1β, and PGE2 compared to untreated control cells, but was not affected in IL-6-treated synovial cells (Fig. 3a). RAMP1 expression increased upon PGE2 stimulation, but remained relatively unchanged in cells treated with TNF-α, IL-1B, and IL-6 (Fig. 3b). No marked differences in CLR expression were detected among the stimulated and untreated synovial cells for any of the examined factors (Fig. 3c).

### Effect of EP1-4 agonists on CGRP and CGRP receptor expression in cultured synovial cells

**Fig. 4:**
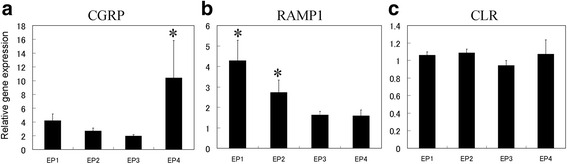
Effects of EP agonists on CGRP and CGRP receptors in synovial cell culture. Real-time polymerase chain reaction analysis for **a** calcitonin gene-related peptide (*CGRP*), **b** receptor activity-modifying protein 1 (*RAMP1*), and **c** calcitonin receptor-like receptor (*CLR*) gene expression in synovial cell culture. Synovial cells were stimulated with 10 μM iloprost (*EP1*), butaprost (*EP2*), sulprostone (*EP3*), or CAY10598 (*EP4*) for 24 h prior to the extraction and analysis of total RNA. All data are presented as the mean ± standard error (*n* = 6). *p < 0.05 compared with the untreated control

**Fig. 5:**
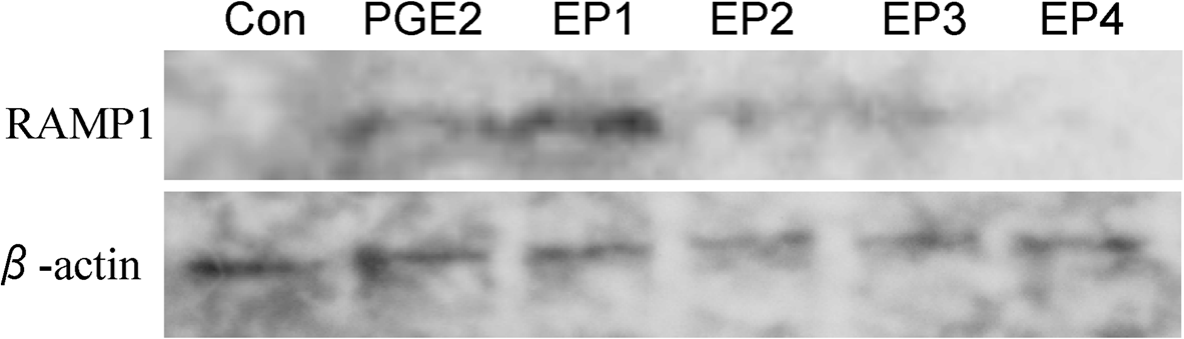
Effects of PGE2 and EP agonists on RAMP1 protein expression in synovial cell culture. Western blotting analysis for RAMP1. Synovial cells were stimulated with 10 μM PGE2, iloprost (*EP1*), butaprost (*EP2*), sulprostone (*EP3*), or CAY10598 (*EP4*) for 24 h prior to protein extraction and analysis of RAMP1 protein

PGE2 acts via four different receptor subtypes: EP1, EP2, EP3, and EP4. Next, the regulation of CGRP and RAMP1 by EPs was investigated using EP1-4 agonists. Real-time PCR analysis revealed that the gene expression of CGRP increased significantly in synovial cells in the presence of exogenously added EP4 agonist compared to untreated control cells (Fig. 4a). The expression of CGRP was not affected in EP1, EP2, and EP3 agonist-treated synovial cells (Fig. 4a). In addition, RAMP1 mRNA expression and protein levels increased significantly in synovial cells treated with exogenously added EP1 and EP2 agonists compared to untreated control cells, but was not affected in EP3 or EP4 agonist-treated synovial cells (Figs. 4b and 5). No differences in CLR expression were detected between the untreated and agonist-treated cells (Fig. 4c).

## Discussion

In the present study investigating the mechanisms underlying the regulation of CGRP in the synovial tissue of knee OA patients, a correlation between CGRP and COX-2 expression was detected, and both CGRP and COX-2 localized in the synovial lining layer. In addition, the treatment of synovial fibroblast cultures with PGE2 and EP4 agonist also stimulated CGRP expression, and PGE2, EP1, and EP2 agonist stimulated RAMP1 expression. Taken together, these findings suggest that the PGE2/EP signaling pathway regulates CGRP/CGRP receptor signaling in synovial fibroblasts.

Recent behavioral studies have reported that CGRP/CGRP receptor signaling is regulated by inflammatory cytokines, growth factors, and PGE2 in neural cells [15, 17], epithelial cells [16], and immune cells [18–20]. In trigeminal ganglion neurons, IL-1β and TNF-α induced CGRP release in [15, 16], and IL-1β additionally promoted COX-2 and PGE2 synthesis, resulting in elevated CGRP release [17]. Liu et al. [16] reported that IL-1β also stimulates CGRP release from human type II alveolar epithelial cells [16]. In the present study, COX-2 expression positively correlated with that of CGRP in synovial tissue, and PGE2, which is the enzymatic product of COX-2, also stimulated CGRP gene expression. In contrast, exogenously added TNF-α and IL-1β stimulated CGRP expression in vitro, but no significant correlation was detected between TNF-α- or IL-1β-induced CGRP mRNA expression levels in the synovial tissue from knee joints of OA patients. In a recent study, Nakata et al. [21] demonstrated that cyclic compressive loading on a 3D-cultured construct of human synovial fibroblasts upregulates PGE2 and COX-2 in the absence of IL-1β or TNF-α stimulation. These results suggest that CGRP may be regulated by PGE2 in an IL-1β- and TNF-α-independent manner in the synovium of OA patients.

RAMP1 is required for the translocation of CLR to the cell surface and also participates in ligand binding and is therefore essential for CGRP receptor signaling [22, 23]. Consistent with these properties, the overexpression of RAMP1 sensitizes vascular smooth muscle cells and trigeminal ganglia neurons to CGRP. A recent study reported that RAMP1 mRNA expression was detected in synovial cell culture isolated from OA patients [5]; however, the underlying regulatory mechanisms controlling this expression were not elucidated. Here, we also detected RAMP1 expression in cultured synovial cells and found that PGE2 regulated not only CGRP expression but also that of the RAMP1 in synovial cells.

PGE2, which is a major pro-inflammatory prostanoid and plays a role in nociceptive processing, acts via four different G-protein-coupled receptor subtypes: EP1, EP2, EP3, and EP4 [24, 25]. EP1 receptors are involved in mechanical sensitization at the spinal cord level. Intrathecal injection of the EP1-selective antagonist ONO-8711 in the carrageenan model of inflammatory pain and oral administration of ONO-8711 in a model of postoperative pain improved mechanical hyperalgesia [26, 27]. The EP2 receptor also contributes to spinal pain sensitization during inflammatory pain states [28, 29]. Furthermore, increased levels of PGE2 upregulates expression of the EP4 receptor subtype in rat sensory dorsal root ganglion (DRG) neurons [30]. Southall et al. [31] reported that the sensitization of sensory neurons is mediated mainly through EP4 receptors and does not proceed via EP3 receptors. Here, we found that the treatment of synovial cell cultures with EP4 agonist led to increased CGRP expression and that the exogenous addition of EP1 and EP2 agonists increased RAMP1 expression. Based on the findings of these previous and present studies, PGE2 appears to regulate CGRP/CGRP receptor signaling through differential EP receptors in the synovium of knee OA patients.

A number of clinical trials have demonstrated that CGRP and CGRP receptor antagonists are efficacious for migraine treatment [32–35]. In addition, humanized antibody against CGRP (LY295174) relieved pain in a rat OA model [6, 7], and clinical trials of anti-CGRP antibody in human OA patients are underway [36]. In the present study, although the relationship between CGRP expression levels and pain in OA patients was not determined, our findings related to CGRP and CGRP receptor regulation in synovial tissue may provide valuable information for developing future pain treatments for OA.

Several limitations of the present study warrant mention. First, the lack of inclusion of a control, non-osteoarthritic patient population is needed to confirm whether CGRP levels are elevated in the synovial tissues of OA patients as compared to non-OA patients. Second, it remains to be determined if the elevation of CGRP levels contributes to OA pain. Third, CGRP mRNA expression and localization of CGRP was examined using real-time PCR and immunohistochemistry, respectively; however CGRP protein concentration was not determined in the synovial tissue or cell culture supernatant. Finally, the effects of CGRP on synovial cells were not evaluated.

## Conclusions

In conclusion, CGRP/CGRP receptor signaling in synovial tissue of OA patients is regulated by PGE2 and PGE2 receptor signaling. The findings presented here may provide useful information for developing therapeutic strategies for managing OA pain.

## Abbreviations

CGRP: Calcitonin gene-related peptide
CLR: Calcitonin receptor-like receptor
COX-2: Cyclooxgenase 2
IL-1β: Interleukin-1β
IL-6: Interleukin-6
NSAIDs: Nonsteroidal anti-inflammatory drugs
OA: Osteoarthritis
PGE2: Prostaglandin E2
RAMP1: Receptor activity-modifying protein 1
TNFα: Tumor necrosis factor-α
